# Optimizing Low–Socioeconomic Status Pregnant Women’s Dietary Intake in the Netherlands: Protocol for a Mixed Methods Study

**DOI:** 10.2196/14796

**Published:** 2020-02-05

**Authors:** Yvette H Beulen, Anouk Geelen, Jeanne H M de Vries, Sabina Super, Maria A Koelen, Edith J M Feskens, Annemarie Wagemakers

**Affiliations:** 1 Health and Society, Department of Social Sciences Wageningen University & Research Wageningen Netherlands; 2 Division of Human Nutrition and Health Wageningen University & Research Wageningen Netherlands

**Keywords:** pregnant women, midwifery, nutrition, social class, healthy diet, health promotion

## Abstract

**Background:**

Although the importance of maternal nutrition is evident, adherence to dietary guidelines is limited in pregnant women, especially in those with a low socioeconomic status. Promotion of a healthy diet in midwifery practice is promising. As prenatal diet affects both maternal and child health, pregnant women are open to dietary changes during this critical transition, and midwives are their first and most important source of information. Unfortunately, nutrition communication by Dutch midwives is limited.

**Objective:**

The objective of this study is to optimize the dietary intake of low–socioeconomic status pregnant women by contributing to the further development and adjustment of a tool or toolbox to support midwives in providing nutrition communication.

**Methods:**

This interdisciplinary, mixed methods study includes 2 phases, in which quantitative and qualitative research are complementary. In phase 1, we will conduct a literature study and interviews to gain insight into midwives’ knowledge, needs, and practice. We will obtain data on the dietary intake of low–socioeconomic status pregnant women and factors influencing this intake from another literature study, an interviewer-administered meal-based food frequency questionnaire, and qualitative interviews with pregnant women. We will identify the availability of suitable tools to improve pregnant women’s dietary intake from the literature, interviews, focus groups, and expert meetings. In phase 2, we shall adapt an existing tool or develop a new tool(box), depending on the results of phase 1, and implement it in 5 midwifery practices. Ultimately, a process evaluation will provide insight into barriers and facilitating factors playing a role in the implementation of the tool(box).

**Results:**

The main outcome of this study will be a tool(box) to optimize the dietary intake of Dutch pregnant women. We anticipate that the developed or adjusted tool(s) will be available in February 2020. After we implement the tool(s) and evaluate the implementation process, the final results should be available by February 2021.

**Conclusions:**

This study is scientifically and socially relevant, as we will study low–socioeconomic status pregnant women’s contextual dietary intake in-depth from an ecological perspective on health. The results obtained will lead to recommendations for multidisciplinary strategies to promote a healthy maternal dietary intake in low–socioeconomic status populations.

**International Registered Report Identifier (IRRID):**

DERR1-10.2196/14796

## Introduction

### Background

The importance of maternal nutrition for optimal fetal development and lifelong population health is increasingly recognized. A healthy dietary intake during pregnancy supports the physical and mental development of the fetus and may prevent congenital malformations, premature birth, and low birth weight or small-for-gestational-age babies [1-4].

Pregnancy provides opportunities to improve dietary intake, as it is a critical transition in the life course during which women consider nutrition important [5]. Nulliparous pregnant women, in particular, show an increased interest in nutrition, although their nutrition-related information-seeking behaviors depend on the time at which they start to feel like a mother [6]. Unfortunately, pregnant women’s adherence to dietary guidelines still appears to be limited [7-9], especially in low–socioeconomic status (SES) populations [10]. Low-SES pregnant women face additional barriers to healthy eating compared with higher-SES groups and experience multiple stressors that may prohibit the instigation and maintenance of a healthy dietary intake [11]. This warrants further study of contextual, behavioral, and psychosocial characteristics of low-SES pregnant women and the associations of these characteristics with dietary intake [11].

The World Health Organization (WHO) claims (pg xii) that interventions improving maternal nutritional status in low-SES populations are among the most effective and sustainable means to achieve positive impacts on health and to reduce health inequalities across generations [4]. In such interventions, diet quality in terms of adherence to dietary recommendations, including supplement use, should be considered, as well as energy balance in relation to gestational weight gain. Successful adoption of a healthy dietary intake depends not only on pregnant women’s nutrition knowledge, but even more so on their ability to decide on, act toward, and sustain a healthy dietary intake given the often stressful or disempowering contexts in which they live [11]. The WHO advocates an inclusive approach to public health and nutrition, such as through support for practitioners to ensure that they understand low-SES pregnant women’s circumstances without stigmatizing them when discussing diet and physical activity [4].

Midwives are an important source of nutrition-related information for most pregnant women in the Netherlands and could potentially play a significant role in improving the dietary intake of low-SES pregnant women [6,12]. Szwajcer et al showed that 80% of Dutch pregnant women were more interested in nutrition information during the first trimester than they were before [6]. Moreover, this study showed that 28% of Dutch women in the first trimester of their pregnancy considered the midwife to be an important channel for pregnancy-related information, including nutrition [6]. Dutch pregnant women considered the midwife to be a trusted source of nutrition information, valued the interactive character of consultations, and perceived the ambiance as pleasant [13]. Dutch midwives independently provide care during normal pregnancy, childbirth, and the early postpartum period. In 2016, 86.8% of pregnant women started consultations in primary prenatal care, and 30.0% of women eventually delivered their child with their primary care midwife. Referral from primary to secondary care during pregnancy or during birth took place in 35.2% and 21.5% of all cases, respectively [14].

Baron et al [12] suggested that a certain amount of proactivity from midwives in providing information may be justified, to increase awareness of beneficial health behaviors and shape positive health behaviors. Although Dutch midwives receive some training in nutrition as part of their education and appear motivated to discuss nutrition in their consultations, their actual nutrition communication appeared limited, was often general in nature, and focused primarily on risks (eg, food safety) and problems (eg, nausea) [12,13]. Midwives seemed to lack essential resources such as expertise, self-efficacy, and time [15,16]. To be more effective, midwives should be able to provide tailored nutrition communication throughout pregnancy and take into account women’s family situation and culture, as well as their current dietary intake [17-19].

Tools could provide support in tailored nutrition communication. Although a variety of tools informing pregnant women about healthy nutrition are available, no evidence-based tools are being used routinely by Dutch midwives to improve pregnant women’s dietary intake. This lack of tools has also been acknowledged by the Royal Dutch Organization of Midwives (*Koninkelijke Nederlandse Organisatie van Versloskundigen*; KNOV), the Dutch Association of Dietitians (*Nederlandse Vereniging van Diëtisten*; NVD), and the Netherlands Nutrition Centre (NNC), and in several scientific papers [20-22]. Tools that could be used may range from pocketbooks to educational videos and workshops [23]. Dietary assessment instruments, including digital ones, might also be helpful for increasing awareness of dietary intake, motivating people to adopt healthy eating habits, or providing support in dietary self-monitoring [24].

Therefore, we will address several omissions in current research and practice in this study. First, we aim to gain insight into the dietary intake of low-SES pregnant women in the Netherlands, including the dynamic interplay between pregnant women and their sociocultural environment. Second, we will study midwives’ current practice and their capability and willingness to provide nutrition communication. Third, we will provide an overview of tools that could support midwives in optimizing pregnant women’s dietary intake. Based on findings on these information gaps, we will either select and adapt or newly develop 1 or more tools fitting best with pregnant women’s and midwives’ needs for implementation in concurrent prenatal practice.

### Objectives and Research Questions

The objective of this study is to develop a tool or toolbox for midwives to promote healthy nutrition among pregnant women, taking into account insights into factors influencing the dietary intake of Dutch low-SES pregnant women. To explore the needs of both pregnant women and midwives and to identify best practices, the main research question is 2-fold:

(1) What is low-SES pregnant women’s contextual dietary intake?

(2) Which tools or methods can midwives use in their daily practice to improve their nutrition communication in order to improve pregnant women’s dietary intake?

In the first part of the project (2.5 years), we will answer 3 research questions and, based on the results, we will develop a tool(box):

(1) What individual (eg, food preferences, information-seeking behavior), interpersonal, and sociocultural (eg, family, social networks, social media) factors drive low-SES pregnant women’s dietary intake? (RQ1)

(2) What resources do midwives need to improve their nutrition communication in order to improve pregnant women’s dietary intake? (RQ2)

(3) What adequate and feasible tools to improve low-SES pregnant women’s dietary intake are available or should be developed? (RQ3)

In the second part of the project (1.5 years), we will implement a newly developed or adapted tool and address a fourth research question will be addressed:

(4) What are the barriers and facilitating factors in relation to using tools or methods to assess and optimize pregnant women’s contextual dietary intake in line with concurrent prenatal care by midwives? (RQ4)

## Methods

### Study Design

We will conduct interdisciplinary, mixed methods research to comprehensively address the research questions. We will collect data in the first part of the research (RQs 1-3) through systematic literature research, interviews with midwives and pregnant women, a diet history questionnaire, focus groups, and expert consultations. In the second part of the study, we will conduct a process evaluation of the implementation of the tool(box) throughout the Netherlands.

Stakeholder involvement is key in this study in order to develop a tool(box) that fits the needs of low-SES pregnant women and is feasible for midwives to use. Midwives, pregnant women, and relevant stakeholders identified in the process will therefore be actively engaged in the research activities. Furthermore, we will involve project partners KNOV, NVD, and NNC in all stages of the research.

### Conceptual Framework

The design of this study is focused on what creates health and well-being rather than on preventing disease. This is encompassed by the concept of salutogenesis, used to explore sources of adaptability and resilience [25]. In this study, we will use 3 building blocks for salutogenic research [26]: (1) taking a holistic orientation to food (nutrition), including physical, mental, and social dimensions of health; (2) supporting a healthful life orientation; and (3) facilitating health-directed learning processes through positive interactions and experiences with food [26].

**Figure 1:**
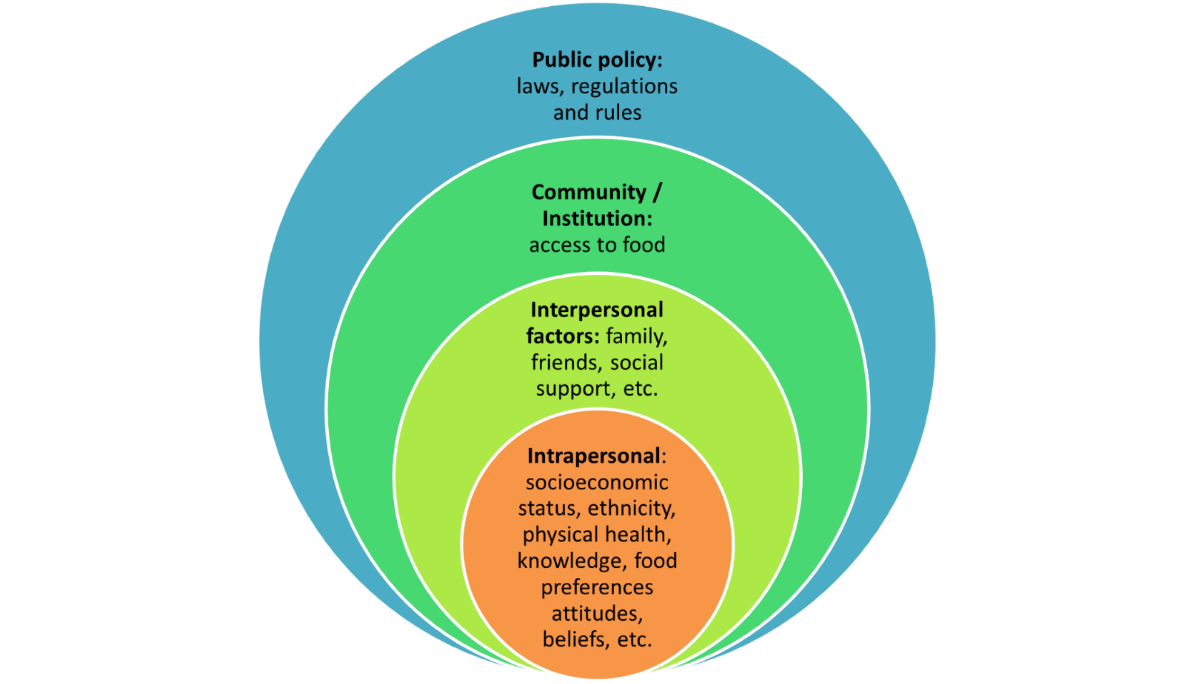
A variation of Bronfenbrenner’s socioecological model adapted to include factors influencing dietary intake based on Fitzgerald and Spaccarotella and Robinson.

**Figure 2:**
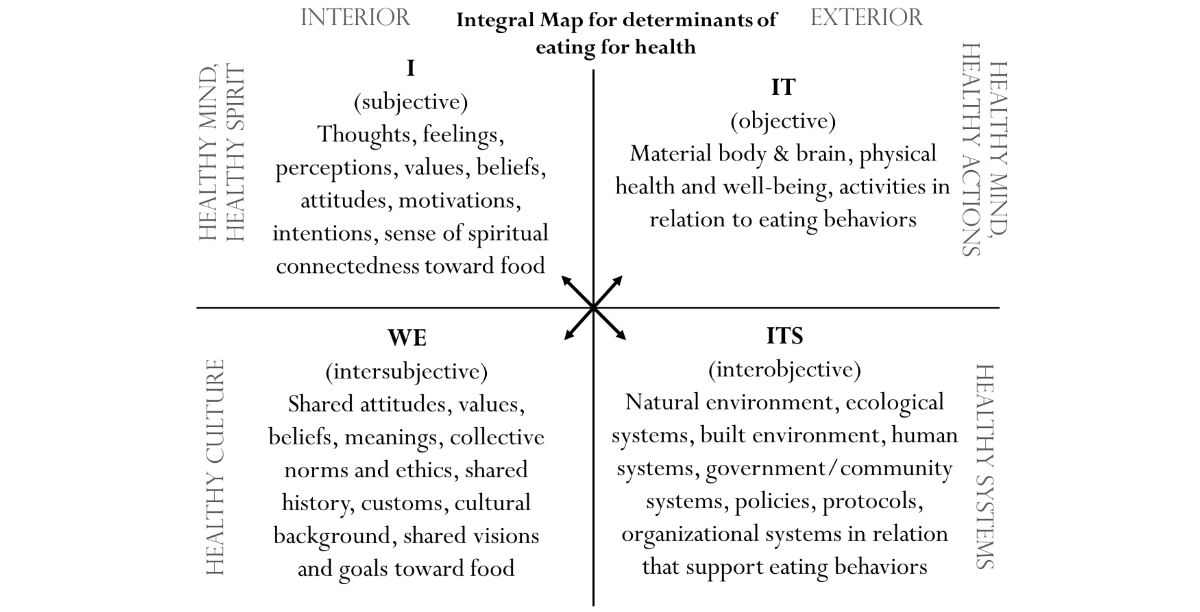
An integral map for integral study of healthful eating by Swan (adapted from Lundy).

The first building block is addressed by means of a socioecological model (Figure 1) and an integral model (Figure 2). Both models provide frameworks for a holistic approach, as they help to elucidate how multiple levels of influence shape a person’s dietary intake [27,28] and to unify multidisciplinary thinking, practice, and evidence gathering [29,30]. The socioecological model allows for categorization of personal, cultural, and environmental factors [31,32], whereas the integral model distinguishes between subjective and objective factors of influence on both the individual and the collective level [30].

The second building block advocates an orientation toward health rather than disease, as conceptualized by the salutogenic model of health. The core constructs of the salutogenic model are sense of coherence and general resistance resources. Sense of coherence comprises the 3 constructs comprehensibility, manageability, and meaningfulness. Comprehensibility encompasses a feeling of confidence that stimuli deriving from one’s internal and external environments are structured, predictable, and explicable; manageability, that resources to meet the demands posed by these stimuli are available; and meaningfulness, that demands are challenges, worthy of investment and engagement [33].

Sense of coherence is inherently related to general resistance resources: resources within an individual or the environment that can be used to counter the stressors of everyday life [34]. From the salutogenic model perspective, it is argued that health promotion activities should focus not only on changing beliefs, knowledge, or intentions, but also on empowering people to mobilize and reflect on resources already available to them [35]. It should be noted that it is not only pregnant women who need to be empowered, but also their midwives. To enable the empowerment process, their relationship should resemble a partnership rather than a traditional hierarchical relationship [36]. In our study, we will use the salutogenic model as a guiding perspective. We will address related concepts of empowerment and reflection in research activities with professionals and pregnant women and in the development or adjustment of a tool(box).

The third and final building block describes health-directed learning processes. Swan’s recommendations include engaging participants and taking into account participants’ social environment or changes in their environment [26]. The best practice framework by Ng and De Colombani comprises these aspects and provides guidance on defining criteria for tools and methods to be used in midwifery practice, with regard to context, process, and outcomes [37]. The framework addresses relevance, community participation, stakeholder collaboration, ethical considerations, and replicability, as well as effectiveness, efficiency, and sustainability. The practice-based evidence available in this framework helps to build on existing tools and practices.

### Study Setting

This study includes low-SES pregnant women and midwives living and working in the Netherlands, as well as stakeholders identified in the process (eg, family members and health professionals, other than midwives). We will collect data on educational level, occupational status of both the participant and, if applicable, her partner, and individual and household income in a general questionnaire to capture the multidimensional nature of SES.

### Recruitment

We will recruit midwives through a combination of convenience sampling and purposive sampling. First, we will approach midwives in the network of project partners, including a midwife, a gynecologist, and the KNOV for participation. Second, we will select midwifery practices located in disadvantaged neighborhoods (both rural and urban) based on postal code, as defined by the Dutch Healthcare Authority [38]. We will approach all midwives selected through the network or through postal code by telephone and email and invite them to participate in an interview and to recruit low-SES pregnant women, or they may opt for just 1 of these activities. We estimate that we will need 20 midwives to reach data saturation. In case we do not reach data saturation, we will conduct more interviews.

We will recruit pregnant women (n=50) through the midwives using a purposive sampling technique. Midwives will receive oral as well as written instructions for the recruitment of pregnant women. We will use highest educational attainment as the primary indicator of SES, as education is a relatively permanent aspect of SES [39] commonly used in nutrition and health research and often included in midwives’ intake questionnaires. Pregnant women will preferably be interviewed as early as possible in pregnancy, as the intervention to be developed will also have to be implemented in the first trimester. All participants taking part in interviews or focus groups should be proficient in Dutch and have a Western dietary pattern. Additionally, we will post flyers and posters in midwifery practice waiting areas to reach pregnant women directly. On these flyers, educational level is communicated positively: “Have you graduated from or are you currently following prevocational or vocational education?:

### Ethics Approval and Consent to Participate

Ethics approval was given by the Social Sciences Ethics Committee of Wageningen University & Research, Wageningen, the Netherlands. The committee thereby declares that the proposal deals with ethical issues in a satisfactory way and that it complies with the Netherlands Code of Conduct for Scientific Practice. Informed consent will be obtained from each participant, after the nature and possible consequences of the study have been explained.

### Data Collection

#### Overview

**Figure 3:**
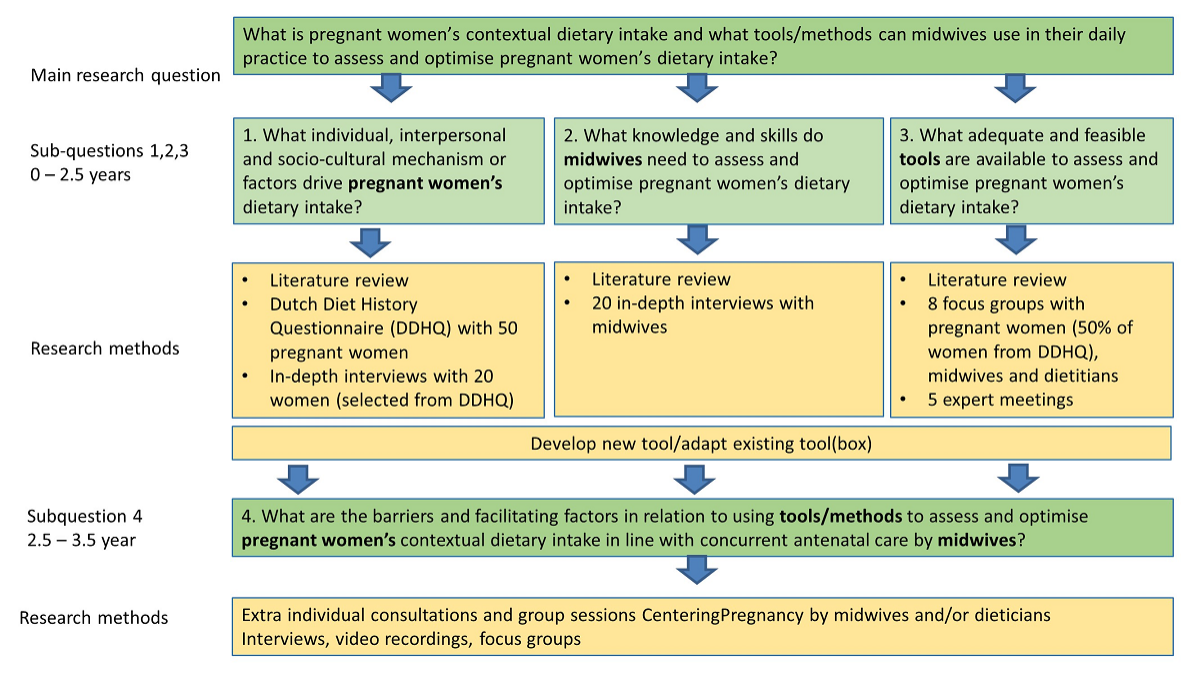
Research questions and participant recruitment per method.

We will collect phase 1 data (RQs 1-3) through systematic literature reviews and interviews and focus groups (n=8) with both pregnant women and midwives. These data will provide insight into factors influencing the dietary intake of low-SES pregnant women (RQ 1); midwives’ current practice, perception of their role, and resources needed to provide nutrition communication (RQ 2); and available tools to optimize pregnant women’s dietary intake, particularly that of low-SES women (RQ 3). Phase 2 will involve implementation of a tool(box), developed or adjusted depending on the results of phase 1, together with all stakeholders, and a process evaluation (RQ 4). Table 1 summarizes the methods and tools for each research question and Figure 3 provides an overview of participant recruitment per research method.

**Table 1:** An overview of research questions, key outcomes, and methods.

Research question	Outcome	Method(s)
What individual, interpersonal, and sociocultural factors drive low–socioeconomic status pregnant women’s dietary intake?	Usual dietary intake	Dutch Diet History Questionnaire
	Factors influencing dietary intake	Literature review, interviews with pregnant women
	Demographics, anthropometrics, health, and lifestyle factors	Questionnaire
What resources do midwives need to improve pregnant women’s dietary intake?	Current nutrition communication by midwives	Interviews with midwives and pregnant women
	Resources needed by midwives to provide nutrition communication	Interviews with midwives
What adequate and feasible tools to improve low–socioeconomic status pregnant women’s dietary intake are available or should be developed?	Needs and expectations with regard to tools	Interviews with midwives and pregnant women, focus groups, expert meetings
	Available tools	Literature review, interviews, expert meetings
What are the barriers and facilitating factors in using tools and methods to assess and optimize pregnant women’s dietary intake in line with concurrent prenatal care by midwives?	Successes and failures in implementation: reach, dose delivered and received, fidelity, context, recruitment, and satisfaction	Pilot study, process evaluation: interviews, video recordings

#### Literature Reviews

We will systematically review the literature for RQs 1 to 3, using the online databases Scopus, Web of Science, and PubMed. The aim of the first literature review is to identify factors influencing pregnant women’s dietary intake. Because few articles focus on pregnant women with a low SES, this review will include articles on pregnant women in general, but will report on differences for SES groups if these are identified in the literature. We will use the second literature review to gain insight into midwives’ perceptions of their role in nutrition communication and resources that they need to optimize pregnant women’s dietary intake. The third and last literature review will identify existing tools and methods to provide nutrition communication to pregnant women. In all reviews, we will exclude articles from low- and middle-income countries, as the results will inform further research activities and the development of a tool(box) for the Dutch setting.

#### Dietary Assessment in Low–Socioeconomic Status Pregnant Women

We will conduct a comprehensive dietary assessment to gain insight into the dietary intake of low-SES pregnant women in the Netherlands. To our knowledge, dietary intake in this specific population has not been studied to date. Obtaining these data will allow us to assess the quality of Dutch low-SES pregnant women’s diet and identify inadequate micronutrient intakes. We will recruit a sample of approximately 50 low-SES pregnant women, based on the research objective, feasibility, and funding [40], and based on previous studies that showed that a sample size of as few as 30 participants can provide a major increase in the width of corrected confidence intervals of associations in a mixed population [41].

To estimate overall usual dietary intake, we will use the diet history method with a reference period of 1 month. To standardize this method, we recently developed a questionnaire, the Dutch Diet History Questionnaire (DDHQ), which is meal based and includes 185 food items. The DDHQ has been developed by trained dietitians and experts in the field of dietary assessment, with a question format based on an existing, validated food frequency questionnaire (FFQ) developed previously at Wageningen University & Research. Food items and portion sizes were adjusted to the target population by analyzing dietary data collected previously among pregnant women [42] and women of reproductive age [43]. A small-scale pilot study (n=7) was conducted to test the face validity and acceptability of the DDHQ in the target group and to improve the comprehensiveness of the food list, as well as its comprehensibility and feasibility. The Dutch FFQ tool was used to generate the computer-based DDHQ [44].

The questionnaire includes an open question for each mealtime to allow for the addition of items missing from the questionnaire. It also includes an open question on supplement use, to assess brand, type, and duration of supplementation. Context is assessed using 3 predefined questions on usual location and timing of meals and social company at each mealtime.

The DDHQ will be administered by trained dietitians to enhance the feasibility of the method for the low-SES study population. Interviewer administration allows for cognitive support in estimating average intake over the reference period, portion sizes, food details, and preparation techniques. Involved dietitians will participate in regular reflection meetings to discuss coding issues, standardize procedures, and minimize interobserver bias. The interviews will last about 1.5 to 2 hours and will take place at the participant’s home or at their midwife’s practice location, depending on each participant’s preference. All participants will receive a gift card for €25 (about US $28).

### Interviews

#### Overview of Semistructured Interviews

We will conduct semistructured interviews to gain further insight into current eating practices of low-SES pregnant women and the need for nutrition advice as perceived by Dutch low-SES pregnant women and midwives. Topic guides will be based on the literature reviews and guided by the salutogenic model of health. We will use appreciative inquiry to create a positive and motivating conversation. Appreciative inquiry relates closely to the concept of salutogenesis and has proved to be effective in organizational life as well as in action research. It builds on participants’ existing strengths and past achievements, rather than on solving a problem [25,45].

We will conduct the interviews both with midwives and with pregnant women until we reach data saturation or have included a maximum of 20 participants. We expect interview duration to be approximately 45 to 60 minutes. Interviews will take place either at the midwife’s practice location or at the participant’s home, according to each participant’s preference. Midwives will receive a financial reward in line with standard hourly wages, and pregnant women will receive another gift card (€15; about US $17).

#### Interviews With Pregnant Women

We will conduct semistructured interviews with pregnant women in a subsample of participants from the DDHQ interviews. All DDHQ interview participants will be invited to participate in an in-depth interview. The interview will take place after the DDHQ interview and not necessarily in the first trimester, as women may be even more experienced or have a clearer view of their needs in retrospect.

We will use visual tools to support communication and represent the data, to summarize themes, and to visualize the data for participants [46]. Prior to the interview, participants will be asked to take a picture of something that is important to them in relation to nutrition during pregnancy. At the start of the interview, the participant will be asked to describe this picture and why it is important to her. This technique is derived from the photovoice method, a participatory action research method. The second visual tool includes picture cards. Cards will be either preprinted based on the topic guide or blank (to be filled out during the interview) and will be used to make a mind map representing factors influencing diet, mentioned throughout the interview. At the end of the interview, this completed mind map will be used for reflection and to determine the most important factors according to the participant.

Two interviewers will be present at each interview. One will primarily be conducting the interview, while the other will be observing, creating the mind map, and complementing the first interviewer by posing additional questions if needed.

#### Interviews With Midwives

We will conduct interviews with midwives to deepen our understanding of midwives’ perceived role and the resources that they need or already use to provide nutrition communication. In addition to the salutogenic model and appreciative inquiry, we will use the 5 A’s construct (or 5 A’s model) in these interviews. The 5 A’s—assess, advise, agree, assist, and arrange—will help to obtain insight into the extensiveness of current nutrition communication in midwifery practice, similar to Van Dillen and colleagues’ method for general practitioners [47,48].

The interviews will be conducted by 2 trained researchers. One of the researchers will primarily conduct the interview, and the other will check whether all questions have been posed and pose additional questions at the end of the interview if needed. We will alternate these roles.

#### Focus Groups

We will organize 2 rounds of semistructured focus groups to complement the individual interviews, as group dynamics may allow for new ideas to arise. The focus groups will be activity oriented where possible, by incorporating choosing, listing, and ranking activities. Such activities can, for instance, be used to test participants’ knowledge and stimulate more in-depth discussions and to make focus groups more enjoyable by doing things rather than just talking [49].

In round 1, we will conduct 2 focus groups of 4 to 12 participants for each study population (pregnant women, midwives, and dietitians) independently. In these focus groups, we will integrate data from the prior individual interviews with pregnant women and midwives and discuss the results (current practice, needs, tool suggestions, and so on) and any inconsistencies. Participants will be a combination of previous interviewees and new participants, to combine those who have already thought of the subject and those with new ideas.

After analyzing the data from the first round of focus groups, in round 2 we will organize 1 or 2 focus groups, attended by pregnant women, midwives, and dietitians combined. In this second round, we will integrate data from the previous focus groups for all participants to work toward a tool(box) together. The mixed focus groups are aimed at facilitating a cocreation process in which important stakeholders together develop the tool(box) based on the research findings.

#### Implementation and Process Evaluation

From the synthesis of the data obtained from the literature and from interviews and focus groups with pregnant women, midwives, dietitians, and experts in the field, we will develop a tool(box) or adjust an existing tool, or both. As the developed tool(box) will be cocreated with all stakeholders and based on the research findings, we cannot yet give an exact description of the tool(box). In addition, it is not yet clear in what setting or settings the tool will be implemented. The developed tool could become part of midwifery practices, be specifically directed at pregnant women, or be implemented by other health care professionals, or a combination thereof. Depending on the cocreation process, we will involve new stakeholders when needed to further develop and implement the tool(box). Experts will be mainly from the Netherlands, but we will also consult experts from countries with similar prenatal care systems.

We will pilot test the tool(box) in real-life practice, specifically for low-SES pregnant women. For the pilot, we will select 5 midwifery practices in different regions of the Netherlands, both urban and rural. We expect that the process evaluation will include approximately 100 individual consultations and 20 CenteringPregnancy group meetings. CenteringPregnancy [50] brings up to 12 pregnant women together for their care and facilitates discussion and activities to address important health topics while leaving room for what is important to the group.

To provide insight into both successes and failures in the implementation of the tool(box), we will conduct a comprehensive process evaluation. In this phase, we may use multiple research methods to understand what works and why. Depending on the eventual tool or tools developed, the methods might include questionnaires, observations through video recording, in-depth interviews, and focus groups. The evaluation will cover common components of process evaluations in public health, such as the proportion of the intended target population participating (ie, reach), how often each part of the tool(box) was delivered by midwives and actually used by pregnant women (ie, dose delivered and received), and the extent to which the intervention was delivered as planned (ie, fidelity). Additionally, it will include environmental and socioenvironmental aspects that may influence implementation (ie, context) and a description of procedures used to approach and attract participants (ie, recruitment) [51]. Lastly, the compatibility of the tool(box) with midwives’ and pregnant women’s needs and expectations (ie, satisfaction) will show whether the involvement of stakeholders in the development of the tool(box) has paid off [52].

### Data Management and Analysis

We will manage data according to Wageningen University’s research data policy, based on the Netherlands Code of Conduct for Scientific Practice and the FAIR (findability, accessibility, interoperability, and reusability) principles. All participants will be assigned a unique study identifier to store data anonymously. We will use the study identifiers to link the quantitative and qualitative data of those participating in both the DDHQ and the in-depth interviews, allowing for investigation of associations between the quality of dietary intake and individual, interpersonal, and sociocultural factors.

 We will audiotape the qualitative research data from the interviews and focus groups with the interviewees’ permission through informed consent, transcribe the audio intelligent verbatim style, and analyze the data by means of thematic analysis [53]. The thematic framework used in the analyses allows for iterative use of both deductive and inductive approaches. Interviews will be coded independently by multiple researchers to reduce interobserver bias and thereby increase the internal validity of the method. We will use ATLAS.ti version 8 software (ATLAS.ti Scientific Software Development GmbH) for these analyses to manage data and optimize transparency.

Quantitative data derived from the DDHQ will be administered and stored in the online Dutch FFQ tool [44], then exported for analysis using SAS version 9.4 statistical software (SAS Institute Inc). Food items in the DDHQ are aggregations of food codes in the Dutch Food Composition Database [54]. Total energy and nutrient intakes will be calculated automatically in the tool by multiplying frequency of intakes by consumed amounts and nutrient composition per item using the same food composition database and standard Dutch food portion sizes. We will present all DDHQ data adjusted for energy to partially account for measurement errors.

## Results

The main outcome of this study will be a tool(box) to optimize dietary intake of Dutch pregnant women. We anticipate that the developed or adjusted tool or tools will be available in February 2020. After we implement the tool(s) and evaluate the implementation process, the final results should be available by February 2021.

## Discussion

### Strengths and Limitations

This study relates to several concurrent health challenges and developments in the Netherlands, such as health inequalities, midwives’ ambition to be strengthened in their role as public health professionals, and translating the first Dutch national dietary guidelines for pregnant women (by an ad hoc committee of the Health Council of the Netherlands) into practice.

This is, to our knowledge, the first time that the dietary intake of specifically low-SES pregnant women in the Netherlands will be studied. Gaining insight into determinants of dietary intake in pregnant women, and specifically low-SES pregnant women, will help to elucidate the factors that contribute to unhealthy habits or—from a more assets-based perspective—the factors that facilitate a healthy dietary intake. By including those pregnant women who would benefit most from nutritional education, this study will address socioeconomic inequalities in health, which are considered unfair and avoidable by, among others, the WHO and governments, in an early stage of life.

The diet history method, applied in an interview by trained dietitians, is the best method for a low-SES target group. However, it is a burdensome method and not well standardized. Therefore, we developed a meal-based questionnaire with food items covering at least 95% of the intake of women of reproductive age. As we will ask additional open questions about the intake of other foods not included in the questionnaire, we expect to cover the complete dietary intake of the women like that captured by the diet history method.

All stakeholders’ perspectives need to be taken into account to generate an evidence base of what works and why in a real-life setting. The participation of multiple stakeholders (pregnant women, midwives, and other health professionals and experts) will be stimulated throughout the research, thereby generating context-sensitive and usable knowledge [55]. We will develop the tool(s) in close collaboration with pregnant women and midwives, KNOV, NVD, and NNC, taking into account their concurrent practices.

All research activities and project meetings conducted in the first phase of the research will contribute to the development of this tool(box) and its implementation and evaluation in the second phase. A limitation of this study is its inability to measure the effectiveness of the developed tool(box) within the scope of the study. An evaluation of the effects on dietary intake, as well as maternal and child health outcomes, could be part of a follow-up study. If the adapted or newly developed tool(box) improves pregnant women’s dietary intake, the impact on perinatal and postnatal outcomes may have health and social benefits, as well as economic benefits.

We will disseminate results to participants who express an interest in this, as inventoried at the end of the interviews. Project partners will be informed on progress, and results will be conveyed orally at least every 6 months by way of a group meeting or individual phone calls. All project partners have furthermore agreed to share the results of the research with their members, for example, through their websites or newsletters. To inform the scientific community, we will disseminate results in scientific journals, as well as at national and international conferences.

### Anticipated Problems

The researchers involved in this study have ample experience with the successful recruitment of low-SES pregnant groups, such as the MetSLIM lifestyle intervention [56], SLIMMER diabetes prevention intervention [57], and Communities on the Move [58]. From these experiences, we have learned that considerable efforts are required to recruit low-SES groups, that too-strict inclusion and exclusion criteria hinder recruitment, and that a personal approach and trust in the recruiter are success factors. Therefore, in this study, we allow for sufficient time for recruitment activities and have budgeted for incentives. We will ask midwives to be gatekeepers in the recruitment of pregnant women, as they have strong trust relationships with their clients. A flexible recruitment protocol will be based on the needs and desires of low-SES pregnant women and their midwives, and incentives, as stated, are budgeted for.

Compared with other countries, in the Netherlands midwives play a central role in maternity care. In general, they are interested in research that will strengthen their profession. Unfortunately, they often experience a lack of time and receive numerous requests to participate in research. We have addressed this problem by holding interviews at their location, by trying to minimize midwives’ time investment in recruitment, and by compensating them financially (based on regular hourly wages).

### Ethical Considerations

Low-SES pregnant women may be considered a vulnerable population for several reasons. First, pregnancy is a time during which women (and their unborn babies) are physically vulnerable. Second, people with a low SES may have problems understanding information. We will instruct interviewers to ensure that participants understand information correctly if the interviewers doubt comprehension and to inform the main researcher about reconsideration of participation. Researchers involved in this project are experienced with research including low-SES groups. The research as a whole is specifically responsive to the health needs and priorities of low-SES pregnant women and ultimately aims to reduce health inequalities.

We are aware of pregnant women’s dependence on midwives and shall therefore emphasize to midwives that all participants should enter the study freely. Participants will not run any physical, social, or political risk by participating in this research.

All participants will be informed about the aims of the research, duration of interviews, data preparation and anonymous data storage, the voluntary nature of participation, and their right to withdraw at any time prior to each research activity. After an opportunity to ask questions, written informed consent will be obtained from each study participant for each research activity. The term low SES, which sounds negative, will not be used in any communication with pregnant women.

### Conclusion

To our knowledge, this will be the first study to address the dietary intake of low–socioeconomic status pregnant women in the Netherlands and to link dietary intake to contextual factors by using an ecological perspective on health. We hope the results obtained will inform multidisciplinary strategies to promote a healthy dietary intake in prenatal care, specifically in low–socioeconomic status populations in developed countries.

## Appendix

Multimedia Appendix 1 Peer-reviewer report #1 from the Edema-Steernberg Foundation.

Multimedia Appendix 2 Peer-reviewer report #2 from the Edema-Steernberg Foundation.

Multimedia Appendix 3 Peer-reviewer report #3 from the Edema-Steernberg Foundation.

## Abbreviations

DDHQ: Dutch Diet History Questionnaire
FFQ: food frequency questionnaire
KNOV: Royal Dutch Organization of Midwives
NNC: Netherlands Nutrition Centre
NVD: Dutch Association of Dietitians
SES: socioeconomic status
WHO: World Health Organization

## References

[1] Abu-Saad K, Fraser D (2010). Maternal nutrition and birth outcomes. Epidemiol Rev.

[2] Christian P, Stewart CP (2010). Maternal micronutrient deficiency, fetal development, and the risk of chronic disease. J Nutr.

[3] Grieger JA, Clifton VL (2014). A review of the impact of dietary intakes in human pregnancy on infant birthweight. Nutrients.

[4] Word Health Organization Regional Office for Europe (2016). Good Maternal Nutrition: The Best Start in Life.

[5] Szwajcer EM, Hiddink GJ, Koelen MA, van Woerkum CMJ (2005). Nutrition-related information-seeking behaviours before and throughout the course of pregnancy: consequences for nutrition communication. Eur J Clin Nutr.

[6] Szwajcer EM, Hiddink GJ, Maas L, Koelen MA, van Woerkum CMJ (2008). Nutrition-related information-seeking behaviours of women trying to conceive and pregnant women: evidence for the life course perspective. Fam Pract.

[7] Blumfield ML, Hure AJ, Macdonald-Wicks L, Smith R, Collins CE (2012). Systematic review and meta-analysis of energy and macronutrient intakes during pregnancy in developed countries. Nutr Rev.

[8] Blumfield ML, Hure AJ, Macdonald-Wicks L, Smith R, Collins CE (2013). A systematic review and meta-analysis of micronutrient intakes during pregnancy in developed countries. Nutr Rev.

[9] Malek L, Umberger W, Makrides M, Zhou SJ (2016). Adherence to the Australian dietary guidelines during pregnancy: evidence from a national study. Public Health Nutr.

[10] Baron R, Manniën J, te Velde SJ, Klomp T, Hutton EK, Brug J (2015). Socio-demographic inequalities across a range of health status indicators and health behaviours among pregnant women in prenatal primary care: a cross-sectional study. BMC Pregnancy Childbirth.

[11] Fowles ER, Bryant M, Kim S, Walker LO, Ruiz RJ, Timmerman GM, Brown A (2011). Predictors of dietary quality in low-income pregnant women: a path analysis. Nurs Res.

[12] Baron R, Heesterbeek Q, Manniën J, Hutton EK, Brug J, Westerman MJ (2017). Exploring health education with midwives, as perceived by pregnant women in primary care: a qualitative study in the Netherlands. Midwifery.

[13] Szwajcer EM, Hiddink GJ, Koelen MA, van Woerkum CMJ (2009). Written nutrition communication in midwifery practice: what purpose does it serve?. Midwifery.

[14] Arns-Schiere AM, van Dijk AE, Dijs-Elsinga J, Henseler A, Hukkelhoven CWPM, de Jonge TE, Stam MC (2018). Perinatale Zorg in Nederland 2016.

[15] Arrish J, Yeatman H, Williamson M (2014). Midwives and nutrition education during pregnancy: a literature review. Women Birth.

[16] McCann MT, Newson L, Burden C, Rooney JS, Charnley MS, Abayomi JC (2018). A qualitative study exploring midwives' perceptions and knowledge of maternal obesity: reflecting on their experiences of providing healthy eating and weight management advice to pregnant women. Matern Child Nutr.

[17] Ferrari RM, Siega-Riz AM, Evenson KR, Moos M, Carrier KS (2013). A qualitative study of women's perceptions of provider advice about diet and physical activity during pregnancy. Patient Educ Couns.

[18] Garnweidner LM, Sverre Pettersen K, Mosdøl A (2013). Experiences with nutrition-related information during antenatal care of pregnant women of different ethnic backgrounds residing in the area of Oslo, Norway. Midwifery.

[19] Brinberg D, Axelson ML (2018). Improving the dietary status of low-income pregnant women at nutritional risk. J Public Policy Mark.

[20] Widen E, Siega-Riz AM (2010). Prenatal nutrition: a practical guide for assessment and counseling. J Midwifery Womens Health.

[21] Derksen M, Wagemakers A (2015). Voedingspatroon inventariseren. Tijdschr Verloskundigen.

[22] Langstroth C, Wright C, Parkington T (2011). Implementation and evaluation of a nutritional screening tool. Br J Midwifery.

[23] Lucas C, Charlton KE, Yeatman H (2014). Nutrition advice during pregnancy: do women receive it and can health professionals provide it?. Matern Child Health J.

[24] Bonilla C, Brauer P, Royall D, Keller H, Hanning RM, DiCenso A (2015). Use of electronic dietary assessment tools in primary care: an interdisciplinary perspective. BMC Med Inform Decis Mak.

[25] Mittelmark MB, Sagy S, Eriksson M, Bauer GF, Pelikan JM, Lindström B, Espnes GA (2017). The Handbook of Salutogenesis. 1st edition.

[26] Swan E (2016). Understanding Healthful Eating From a Salutogenic Perspective [dissertation].

[27] McLeroy KR, Bibeau D, Steckler A, Glanz K (1988). An ecological perspective on health promotion programs. Health Educ Q.

[28] National Cancer Institute (2005). Theory at a Glance: A Guide for Health Promotion Practice.

[29] Hanlon P, Carlisle S, Reilly D, Lyon A, Hannah M (2010). Enabling well-being in a time of radical change: integrative public health for the 21st century. Public Health.

[30] Lundy T (2010). A paradigm to guide health promotion into the 21st century: the integral idea whose time has come. Glob Health Promot.

[31] Fitzgerald N, Spaccarotella K (2009). Barriers to a healthy lifestyle: from individuals to public policy—an ecological perspective. J Extension.

[32] Robinson T (2008). Applying the socio-ecological model to improving fruit and vegetable intake among low-income African Americans. J Community Health.

[33] Antonovsky A (1987). Unraveling the Mystery of Health. How People Manage Stress and Stay Well.

[34] Lindström B, Eriksson M (2010). Salutogenesis – an introduction. Hitchhiker's Guide to Salutogenesis: Salutogenic Pathways to Health Promotion.

[35] Super S, Wagemakers MAE, Picavet HSJ, Verkooijen KT, Koelen MA (2016). Strengthening sense of coherence: opportunities for theory building in health promotion. Health Promot Int.

[36] Koelen MA, Lindström B (2005). Making healthy choices easy choices: the role of empowerment. Eur J Clin Nutr.

[37] Ng E, de Colombani P (2015). Framework for selecting best practices in public health: a systematic literature review. J Public Health Res.

[38] Nederlandse Zorgautoriteit (2016). Prestatie – en Tariefbeschikking Verloskunde – TB/REG-17625-02.

[39] Kunst AE, Dalstra JAA, Bos V, Mackenbach JP, Otten FWJ, Geurts JJM (2005). Ontwikkeling en toepassing van indicatoren van sociaal-economische status binnen het Gezondheidsstatistisch Bestand.

[40] Food and Agriculture Organization (2018). Dietary Assessment: A Resource Guide to Method Selection and Application in Low Resource Settings.

[41] Willett W (2013). Nutritional Epidemiology. 1st edition.

[42] Looman M, van den Berg C, Geelen A, Samlal RAK, Heijligenberg R, Klein Gunnewiek JMT, Balvers MGJ, Leendertz-Eggen CL, Wijnberger LDE, Feskens EJM, Brouwer-Brolsma EM (2018). Supplement use and dietary sources of folate, vitamin D, and n-3 fatty acids during preconception: the GLIMP2 study. Nutrients.

[43] Brouwer-Brolsma EM, van Lee L, Streppel MT, Sluik D, van de Wiel AM, de Vries JHM, Geelen A, Feskens EJM (2018). Nutrition Questionnaires plus (NQplus) study, a prospective study on dietary determinants and cardiometabolic health in Dutch adults. BMJ Open.

[44] Molag M (2010). Towards Transparent Development of Food Frequency Questionnaires [dissertation].

[45] Cooperrider DL, Whitney D, Stavros JM (2005). Appreciative Inquiry Handbook. 2nd edition.

[46] Glegg SMN (2019). Facilitating interviews in qualitative research with visual tools : a typology. Qual Health Res.

[47] Whitlock EP, Orleans CT, Pender N, Allan J (2002). Evaluating primary care behavioral counseling interventions: an evidence-based approach. Am J Prev Med.

[48] van Dillen SME, van Binsbergen JJ, Koelen MA, Hiddink GJ (2013). Nutrition and physical activity guidance practices in general practice: a critical review. Patient Educ Couns.

[49] Colucci E (2007). “Focus groups can be fun”: the use of activity-oriented questions in focus group discussions. Qual Health Res.

[50] van Zwicht B, Crone M, van der Pal-de Bruin K (2013). Centering pregnancy in Nederland. Prenatale Zorg.

[51] Linnan L, Steckler A, Linnan L, Steckler A (2002). Chapter one. Process evaluation for public health interventions and research. An overview. Process Evaluation for Public Health Interventions and Research.

[52] Saunders RP, Evans MH, Joshi P (2005). Developing a process-evaluation plan for assessing health promotion program implementation: a how-to guide. Health Promot Pract.

[53] Braun V, Clarke V (2006). Using thematic analysis in psychology. Qual Res Psychol.

[54] (2019). Dutch food composition database.

[55] Wagemakers A (2010). Community Health Promotion. Facilitating and Evaluating Coordinated Action to Create Supportive Social Environments [dissertation].

[56] Bukman AJ, Teuscher D, Meershoek A, Renes RJ, van Baak MA, Feskens EJ (2017). Effectiveness of the MetSLIM lifestyle intervention targeting individuals of low socio-economic status and different ethnic origins with elevated waist-to-height ratio. Public Health Nutr.

[57] Duijzer G, Haveman-Nies A, Jansen SC, ter Beek J, Hiddink GJ, Feskens EJM (2014). SLIMMER: a randomised controlled trial of diabetes prevention in Dutch primary health care: design and methods for process, effect, and economic evaluation. BMC Public Health.

[58] Herens M (2016). Promoting Physical Activity in Socially Vulnerable Groups. A Mixed Method Evaluation in Multiple Community-Based Physical Activity Programs [dissertation].

