# Supplementary material for: Optimizing Low–Socioeconomic Status Pregnant Women’s Dietary Intake in the Netherlands: Protocol for a Mixed Methods Study
Source: JMIR Res Protoc. 2020 Feb 5;9(2):e14796. doi: 10.2196/14796 (PMC7055783; doi:10.2196/14796)
Supplement: Multimedia Appendix 1 [file resprot_v9i2e14796_app1.pdf]

REPLY FORM

Each aspect is described in terms of a range between outstanding (1) and unsatisfactory (4), with two intermediate ratings in between. Please tick appropriate column for each aspect. More detailed remarks can be made on page 2.

	1	2	3	4	
SCIENTIFIC QUALITY	**Project proposal**				
Clearly written		X			Unclear
	**Originality**				
High		X			Low
	**Methodological approach**				
Strong			X		Weak
	**Scientific perspectives**				
Of great interest		X			Of little interest
FEASIBILITY	**Work plan**				
Feasible in four years	X				Feasibility doubtful
	**Use of existing knowledge**				
Ample	X				Poor
	**Equipment / techniques**				
Appropriate			X		Poor
	**Supervision**				
Sufficient	X				Insufficient
	**Co-operation with others**				
Adequate	X				Insufficient
CONCLUSION	**Quality and feasibility**				
High		X			Modification needed

Referee's detailed comments:

This is an interesting proposal about an important aspect of maternal and child health.
My main concern is about the proposed methodology which seems entirely qualitative in nature. I am not familiar with the PhD requirements of Wageningen University, but for a four-year PhD study I would expect at least some quantitative elements.
The overall goal of the study is to contribute to the improvement of the nutritional status of low SES pregnant women, by the development of tools or methods that midwives can use to assess and optimise dietary intake of their clients. To develop these tools or methods more information is needed about pregnant women's dietary intake and about relevant knowledge and skills of midwives.
Appendix 3 shows the research methods: literature study, interviews (individual and in focus-groups) and expert consultation in the first part of the study, interviews, video recordings and focus groups in the second part. However, in the method section questionnaires are mentioned as well, without any explanation about what kind of questionnaire, for whom they are supposed to be and when they will be used. On the other hand, video recordings are mentioned in appendix 3, but are not explained in the text. It is not clear what will be recorded, how many recordings will be made and how those recordings will be analysed.
Based on Appendix 3 and on the Work-plan provided in the text, I conclude that all data collected will be qualitative. However, in the Research Data Management Plan frequency tables and regression models are promised. How do you propose to use regression models on these qualitative data? Frequency tables are possible, but in my view only to present background information of the respondents. Presenting outcome information from interviews and focus groups in frequency tables will give the impression that these data are representative for the study population, which you can't be sure they are. Although a footnote in the work-plan says that the exact number of interviews will depend on data saturation, that does not mean the results can be extrapolated beyond the respondents. Data saturation means that you probably have found most of the important differences and peculiarities within the group you are studying, but it says nothing about how often these peculiarities occur within the population.
In the work-plan and in appendix 3 20 interviews with midwives are mentioned, 20 with pregnant women and their partners and 10 focus groups. According to the appendix midwives are also included in the focus groups, but that is not mentioned in the text or the work-plan.
It is not clear to me what the diet history method is, how structured or open this method is and how detailed the information is that will be collected by the dietitians. The target is at least 50 women, but it is unclear when, how and by whom will be decided that 50 is or is not enough.
In the Summary and in the Methodology is mentioned that: 'it is expected that 100 extra individual consultations and 20 extra Centering Pregnancy group meetings will be organised.' I understand the second part of the study is not very detailed yet, but when the word 'extra' is used, I assume that individual consultations and CP-group meetings have already been included in the study protocol, but that is not the case. And I don't see what aspect of an individual consultation is part of the study project, what data will be collected during those consultations or group meetings.

One other remark: about the supposed innovative character of the study. In the Summary is written: 'Innovatively, midwives, dietitians and pregnant women are engaged in research activities …', but for me it is unclear what is innovative about this proposal. Midwives and pregnant women are interviewed, they are subjects in the study, but that is not innovative. Both groups will be participating in a test phase in the second part of the study, but that is not innovative either. How else would you test new tools? Dietitians have a different role, as research assistants, bur what is innovative about that?

Conclusion: more information is needed about the data collection and analyses procedures. And the innovative character of the study needs a better explanation.

GUIDELINES for the review of a PhD project proposal

You are kindly requested to answer the following questions in your appraisal of the proposal:

SCIENTIFIC QUALITY

**Project proposal**	Are the objectives, scientific and social importance, hypotheses, methodology and work plan clearly described?
**Originality**	Does the proposal show high scientific originality as should be expected from a PhD project?
**Methodological approach**	Is the methodological approach strong and up-to-date?
**Scientific perspectives**	Does the proposal have interesting scientific relevance and perspectives for the own discipline, other disciplines and/or in an interdisciplinary way?

FEASIBILITY

**Work plan**	Can the proposed research *and* the writing of an approved draft of the dissertation be completed in the four-year period a PhD student is appointed?<br>Take into account that 10-15% of that time is spent to attend courses, seminars, symposia etc. and a maximum of 10% on teaching duties.
**Use of existing knowledge**	Does the research plan show that ample use was and will be made of existing knowledge and expertise?
**Equipment / techniques**	Is the proposed use of equipment and techniques appropriate and up-to-date?
**Supervision**	Is the supervision qualitatively and quantitatively sufficient?
**Co-operation with others**	Is the envisaged co-operation with others adequate, or have any necessary institutions been left out?

CONCLUSION

**Quality and feasibility**	Are both the scientific quality and feasibility appropriate for a PhD project, or would you recommend modification of the project proposal?

---

[1] The appointment of a PhD-student is for a period of 4 years. A maximum of 15% of the total contract period may be spent to attend educational activities (courses, seminars, congresses, etc.)
