# Supplementary material for: Optimizing Low–Socioeconomic Status Pregnant Women’s Dietary Intake in the Netherlands: Protocol for a Mixed Methods Study
Source: JMIR Res Protoc. 2020 Feb 5;9(2):e14796. doi: 10.2196/14796 (PMC7055783; doi:10.2196/14796)
Supplement: Multimedia Appendix 2 [file resprot_v9i2e14796_app2.pdf]

REPLY FORM

Each aspect is described in terms of a range between outstanding (1) and unsatisfactory (4), with two intermediate ratings in between. Please tick appropriate column for each aspect. More detailed remarks can be made on page 2.

	1	2	3	4	
SCIENTIFIC QUALITY	**Project proposal**				
Clearly written	√				Unclear
	**Originality**				
High	√				Low
	**Methodological approach**				
Strong	√				Weak
	**Scientific perspectives**				
Of great interest	√				Of little interest
FEASIBILITY	**Work plan**				
Feasible in four years		√			Feasibility doubtful
	**Use of existing knowledge**				
Ample	√				Poor
	**Equipment / techniques**				
Appropriate	√				Poor
	**Supervision**				
Sufficient	√				Insufficient
	**Co-operation with others**				
Adequate	√				Insufficient
CONCLUSION	**Quality and feasibility**				
High	√				Modification needed

Referee’s detailed comments:
SCIENTIFIC QUALITY

The Project proposal addresses an issue of utmost importance to human health, nutrition in pregnancy. The objectives are clearly stated, with a goal to improve the nutritional status of low SES pregnant by working with mid-wives to ascertain contextual factors related to dietary intake and appropriate ways I which mid-wives can assist pregnant to improve intakes. The project has high social importance. The hypotheses will be tested under 4 sub0questions. The methodology and work plan are clearly described?

Originality The proposal shows scientific originality and is consistent with what is expected from a PhD project? Most research focusses on interventions outside health services, so this has strong applicability to maternity healthcare practice

Methodological approach The methodological approach is strong but I suggest that at least one of the reviews be conducted as a systematic review, to ensure rigor in the evaluation of the scientific quality of the literature to date and also to have an up-to-date review published from the thesis work plan?

Scientific perspectives The proposal has scientific relevance perspectives for the applicants own disciplines and for other disciplines and also in an interdisciplinary way? I think an improvement could be made by giving further consideration as to how dietitians can work *with* midwives in developing models of care that could capitalise of the nutrition expertise, rather than rely of midwives have to develop nutrition expertise. For example the midwives could be responsible for nutrition screening and then reinforcement of nutrition messages relevant to pregnant women.

FEASIBILITY

Work plan I think the proposed research *and* the writing of the dissertation be completed in the four-year period a PhD student is appointed, but this will be dependent on receiving timely feedback from all supervisors involved in the thesis work plan and timely ethics approvals.

Use of existing knowledge Yes the research plan will use existing knowledge and expertise? I suggest that at least one of the reviews be conducted as a systematic review.

Equipment / techniques Yes the proposed techniques are appropriate and up-to-date?

Supervision The supervision team is excellent and has the expertise needed for the project.

Co-operation with others The project component that I think should be considered more is the inclusion of the dietitian professional body in the nutrition assessment and education of the mid-wives in terms of capitalising on the nutrition expertise already available in the healthcare system.

CONCLUSION

Quality and feasibility Both the scientific quality and feasibility are appropriate for a PhD project. It is a lot of work, but I note you have a four year rather than a three year time frame?

GUIDELINES for the review of a PhD project proposal

You are kindly requested to answer the following questions in your appraisal of the proposal:

SCIENTIFIC QUALITY

**Project proposal**	Are the objectives, scientific and social importance, hypotheses, methodology and work plan clearly described?
**Originality**	Does the proposal show high scientific originality as should be expected from a PhD project?
**Methodological approach**	Is the methodological approach strong and up-to-date?
**Scientific perspectives**	Does the proposal have interesting scientific relevance and perspectives for the own discipline, other disciplines and/or in an interdisciplinary way?

FEASIBILITY

**Work plan**	Can the proposed research *and* the writing of an approved draft of the dissertation be completed in the four-year period a PhD student is appointed?<br>Take into account that 10-15% of that time is spent to attend courses, seminars, symposia etc. and a maximum of 10% on teaching duties.
**Use of existing knowledge**	Does the research plan show that ample use was and will be made of existing knowledge and expertise?
**Equipment / techniques**	Is the proposed use of equipment and techniques appropriate and up-to-date?
**Supervision**	Is the supervision qualitatively and quantitatively sufficient?
**Co-operation with others**	Is the envisaged co-operation with others adequate, or have any necessary institutions been left out?

CONCLUSION

**Quality and feasibility**	Are both the scientific quality and feasibility appropriate for a PhD project, or would you recommend modification of the project proposal?

---

[1] The appointment of a PhD-student is for a period of 4 years. A maximum of 15% of the total contract period may be spent to attend educational activities (courses, seminars, congresses, etc.)
