# Supplementary material for: Optimizing Low–Socioeconomic Status Pregnant Women’s Dietary Intake in the Netherlands: Protocol for a Mixed Methods Study
Source: JMIR Res Protoc. 2020 Feb 5;9(2):e14796. doi: 10.2196/14796 (PMC7055783; doi:10.2196/14796)
Supplement: Multimedia Appendix 3 [file resprot_v9i2e14796_app3.pdf]

REPLY FORM

Each aspect is described in terms of a range between outstanding (1) and unsatisfactory (4), with two intermediate ratings in between. Please tick appropriate column for each aspect. More detailed remarks can be made on page 2.

	1	2	3	4	
SCIENTIFIC QUALITY	**Project proposal**				
Clearly written	x				Unclear
	**Originality**				
High		x			Low
	**Methodological approach**				
Strong	x				Weak
	**Scientific perspectives**				
Of great interest		x			Of little interest
FEASIBILITY	**Work plan**				
Feasible in four years	x				Feasibility doubtful
	**Use of existing knowledge**				
Ample	x				Poor
	**Equipment / techniques**				
Appropriate	x				Poor
	**Supervision**				
Sufficient	x				Insufficient
	**Co-operation with others**				
Adequate	x				Insufficient
CONCLUSION	**Quality and feasibility**				
High	x				Modification needed

Referee's detailed comments:

Scientific quality and innovativeness of research plan

It is widely acknowledged that exposures already in fetal life may influence health and health inequalities throughout the lifespan. However, there is a lack of insight into health promotion potential and barriers during pregnancy. This PhD project has the potential to provide much new insight into the issue and has the potential for significant outcomes. The planned research combines scientific knowledge with expertise and experiences from midwives, dietitians, pregnant women and their partners. The project has many merits: one is the application of the socio-ecological model as a guiding principle for data collection and analysis. Another merit is the relatively focused approach (pregnant women's dietary intake). A third merit is engaging and benefitting stakeholders, especially ones with low SES. A fourth merit is two stage approach: First, the research group i) recognizes mechanisms and/or factors driving pregnant women's dietary intake, ii) knowledge and skills of midwives in terms of assessing and optimizing the dietary intake, and iii) tools available for dietary assessment. Second, the tools and methods are further developed for low SES pregnant women and those with a non-Western dietary pattern. It is also a merit that the researchers plan to combine qualitative and quantitative methods and data.

Although there is a good rationale for the proposal and the need for such research, awareness of potential difficulties is not spelled out enough. The biggest challenge might be faced when reaching and recruiting pregnant women with low SES. It is also not clear if tools and methods used in the second part are aimed to act across multiple levels of the socio ecological model.

Feasibility of research plan
The supervisors combine excellent complementary skills related to the topics and methods involved. The host institution has excellent infrastructure and expertise, and the collaborating partners have strong track records in their respective fields. The entire research plan appears ambitious yet realistic and feasible. The proposed research and writing can be completed in the four-year period.

Conclusion
This is important research in an understudied area of public health. The overall goal of the project to improve the nutritional status of low SES pregnant women is ambitious yet feasible. The research questions are justified, all of them with the potential to provide new insight. The study has potential to impact practices and to provide evidence based tools and methods to optimize pregnant women's dietary intake.
